# Does BMP2 play a role in the pathogenesis of equine degenerative suspensory ligament desmitis?

**DOI:** 10.1186/s13104-018-3776-9

**Published:** 2018-09-18

**Authors:** Madeline Young, Olaniyi Moshood, Jian Zhang, Carolyn A. Sarbacher, P. O. Eric Mueller, Jaroslava Halper

**Affiliations:** 1 Department of Pathology, College of Veterinary Medicine, The University of Georgia, Athens, GA 30602 USA; 2 Department of Large Animal Medicine, College of Veterinary Medicine, The University of Georgia, Athens, GA 30602 USA; 3 Department of Pathology, College of Veterinary Medicine, AU/UGA Medical Partnership, The University of Georgia, Athens, GA 30602 USA

**Keywords:** Equine degenerative suspensory ligament desmitis, Proteoglycans, BMP2

## Abstract

**Objective:**

Horses afflicted with degenerative suspensory ligament desmitis (DSLD) suffer from progressive leg pain and lameness without history of trauma. DSLD is a systemic disorder caused by abnormal accumulation of proteoglycans in many connective tissues. One proteoglycan found in higher quantities in DSLD is decorin. The accumulated decorin has an abnormally glycosylated glycosaminoglycan chain in DSLD. In addition to acellular accumulations of proteoglycans foci of active fibroblasts/tenoblasts were observed in some tendons and suspensory ligaments (SLs) from DSLD cases We have hypothesized that this represents an early event in DSLD and that production of chondrogenic growth factors, such as BMP2, and/or enzyme participating in glycosylation of glycosaminoglycans is a major factor in initiation and progression of DSLD.

**Results:**

Using immunohistochemistry we have identified BMP2 in these cellular foci, indicating association with proteoglycan production, but not in other cells in the tendon and SLs. In contrast, very little staining for TGFβ and dermatan sulfate epimerase, an enzyme involved in glycosylation of glycosaminoglycan chains, was observed in these foci and other cells in both control and DSLD-affected tendons and SLs. Our data support our hypothesis that chondrogenic growth factors may be responsible, at least in part for progression of DSLD in horses.

## Introduction

Equine Degenerative Suspensory Ligament Desmitis (DSLD) was identified first in Peruvian Paso horses [1]. Though this breed is most commonly affected, DSLD has been diagnosed with increasing frequency in other horse breeds including Quarter horses, Warmbloods, Arabians and others [2, 3]. Affected horses present typically with progressive bilateral or quadrilateral lameness, dropped fetlocks and painful lower extremities with no evidence or history of trauma. The diagnosis of DSLD is based on health history and presentation, pain upon palpation of the suspensory ligament (SL) and diffuse enlargement of SL on physical examination. Ultrasonography of the limb reveals SL enlargement with a hypoechoic, irregular fiber pattern. There is no cure for the disease and care involves supportive and palliative measures such as controlled exercise, corrective shoeing and pain relief [4]. There is no known way to stop the progression of DSLD with many of these horses requiring humane euthanasia. Little is known about the specific pathogenesis of DSLD making prevention, diagnosis and effective treatment of the affected horses difficult. Hereditary component has been suspected because of frequent familial appearance of DSLD during a common age for breeding, the potential for transmitting DSLD to offspring is high [2–4].

Though considered originally a disorder of collagen limited to SLs of lower extremities, later on we characterized DSLD as a systemic disorder caused by abnormal accumulation of proteoglycans in a wide variety of connective tissues [3]. This accumulation is particularly damaging to weight-bearing tendons and ligaments of equine extremities [5]. Our lab was the first to bring attention to proteoglycan abnormalities as a potential mechanism [3, 5]. At least one of the tendon/ligament proteoglycans, decorin, is abnormal in horses with DSLD, with chondroitin sulfate replacing the normal dermatan sulfate due to a defect in glycosylation of the glycosaminoglycan (GAG) chain [5]. Decorin plays an essential role in regulating collagen fibrillogenesis, and spatial organization of collagen fibrils and any abnormality in decorin could lead to abnormal collagen fibrils resulting in decreased biomechanical strength [6–8].

As part of our ongoing study of pathogenesis of DSLD we examined the presence of a chondrogenic growth factor(s) in DSLD using immunohistochemistry. The best characterized chondrogenic factors are transforming growth factor β-3 (TGFβ-3), and bone morphogenetic proteins 2 and 7 (BMP2 and BMP7) [9–11]. In addition, of the many enzymes involved in synthesis of GAG chains attached to core proteins of proteoglycans that may play another role in the development of DSLD dermatan sulfate epimerase is of particular interest because of its role in synthesis of dermatan sulfate chains [12–14].

## Main text

### Methods

#### Subjects and tissue collection

Superficial and deep digital flexor tendons, and SLs were collected from extremities of horses with and without DSLD (Table 1). The horses were either donated for research and euthanized, or were undergoing necropsy for diagnostic purposes. Euthanasia was performed in accordance with institutional guidelines and consisted of intravenous overdose of sodium pentobarbital solution at a dose of > 87 mg/kg/IV. Some tendon samples were submitted to us for diagnostic purposes.

**Table 1:** Subject characterization and results of immunohistochemistry

Case	Tissue	BMP2 cell	BMP PG	Epimerase	TGFβ	Age, breed, sex
H1	SDFT, SL	+	N/A	++	+/−	9 year PP male
H2	SDFT, SL	++/+	N/A	+	−	15 year PP mare
H3	2 SLs	++	N/A	+/−	+/−	18 year QH mare
H4	SDFT, SL	++	N/A	−	ND	1.5 year PP male
H5	SL	++	+	−/+	ND	7 year PP mare
H6	SDFT, DDFT	++/+	−	++/+	ND	5 year PP male
H7	SDFT	++++/+++	+	++	+/−	17 year WB gelding
H8	2 SLs	++++	+/++	+/−	+	4 year Arab gelding
H9	SFDT, DDFT, SL	+++/++	+	+/−	+/−	6 year Amer Saddle male
H10	SL	−	++/−	+/−	−	Unknown
H11	SL	+	+	−/+	++	11 year QH mare
H12	SDFT	+++	−	++	−	9 year QH gelding
H13	2 SDFTs DDFT, SL	++/+ +/−	ND	++ +/−	ND	16 year PP female
H14	SL	+++	++/+	+/−	ND	15 year holsteiner gelding
D1	DDFT, SL	++	+/−	+/−	++/+	13 year PP mare
D2	SDFT, DDFT, SL	++	+/−	+/−	ND	24 year PP male
D3	SDFT, DDFT	+/−	+/−	+/−	ND	14 year PP mare
D4	LF	++/+	+	+/−	ND	17 year PP mare
D5	2 SDFTs DDFT, SL	+++	N/A	ND	+/−	fetus PP (of D4)
D6	DDFT, SL	+++	++/+	++/+	++/−	18 mo TB female
D7	DDFT, SL SDFT	++/+ +	+	ND	ND	20 year PP male
C1	SDFT, DDFT, SL	−	N/A	+/−	+/−	33 year PP stallion
C2	DDFT, SL	+/−	N/A	+/−	+/−	8 year QH female
C3	DDFT, SL	+/−	N/A	+/−	+/−	5 mo Percheron male
C4	SDFT, DDFT, SL	++/+	N/A	+/−	ND	31 year PP mare
C5	SDFT, DDFT, SL	+/−	N/A	+/−	ND	32 year PP mare

*H* DSLD horse with many active foci or hypercellularity, limited PGs, *D* DSLD horse with mostly PGs and limited hypercellularity, *C* control horse, *PP* Peruvian Paso, *QH* quarter horse, *TB* thoroughbred, *SDFT* superficial deep digital flexor tendon, *DDFT* deep digital flexor tendon, *SL* suspensory ligament, *RH* right hind limb

#### Immunohistochemistry

Standard hematoxylin and eosin staining of tissue sections was used for initial evaluation and diagnosis. Immunohistochemistry was performed on deparaffinized slides using a standard protocol. Endogenous peroxidase was quenched with 3% H_2_O_2_ for 10 min at room temperature (RT). After washing with a buffer nonspecific sites were blocked with a universal blocking agent (Biogenex Laboratories, Fremont, CA, USA). Following antibodies were used: rabbit polyclonal antibody sc-146 (Santa Cruz Biotechnology, Santa Cruz, CA, USA) to detect TGFβ-1 at dilution 1:500 for 2 h at 37 °C or overnight at 4 °C; anti-BMP2 rabbit polyclonal antibody ab82511 (Abcam, Cambridge, UK) was used at dilution 1:1000 for 2 h at 37 °C or overnight at 4 °C, and rabbit polyclonal anti-glucuronic acid epimerase antibody (Novus Biologicals, Littleton, CO, USA) was used at 1:200 dilution for 1 h at RT. After incubation with a primary antibody the slides were incubated with secondary biotinylated antirabbit antibody for 2 h at RT before Avidin Biotin complex (Elite PK-6100 Standard kit) (Vector Burlingame, CA, USA) was applied for 1 h at RT. Antibody-antigen complexes were detected with 3,3′-diaminobenzidine (DAB) Peroxidase (HRP) Substrate Kit (with Nickel, SK-4100), also from Vector Laboratories. Slides were counterstained with hematoxylin. Primary antibodies were omitted in control slides. The staining was evaluated primarily for intensity and presence in fibroblasts or tenoblasts (from + to +++) rather than distribution and range in extracellular tissue because collagen and even more proteoglycans exhibit strong background staining.

### Results

#### Subjects

Tendons and SLs were obtained from 21 horses from DSLD and from 5 control horses without DSLD (Table 1). The DSLD group was divided in two sub-groups, one consisting of 14 subjects (H1-H14) with prominent hypercellular foci in their tendons and SLs, whereas tendons and SLs from 7 horses with prevalence of large proteoglycan collections were in included in the second sub-group (D1-D7. The horses ranged from a fetus (D5) to 33 years (C1, C4 and C5)) in age with both sexes and several breeds included (Table 1).

#### Histopathology

**Fig. 1:**
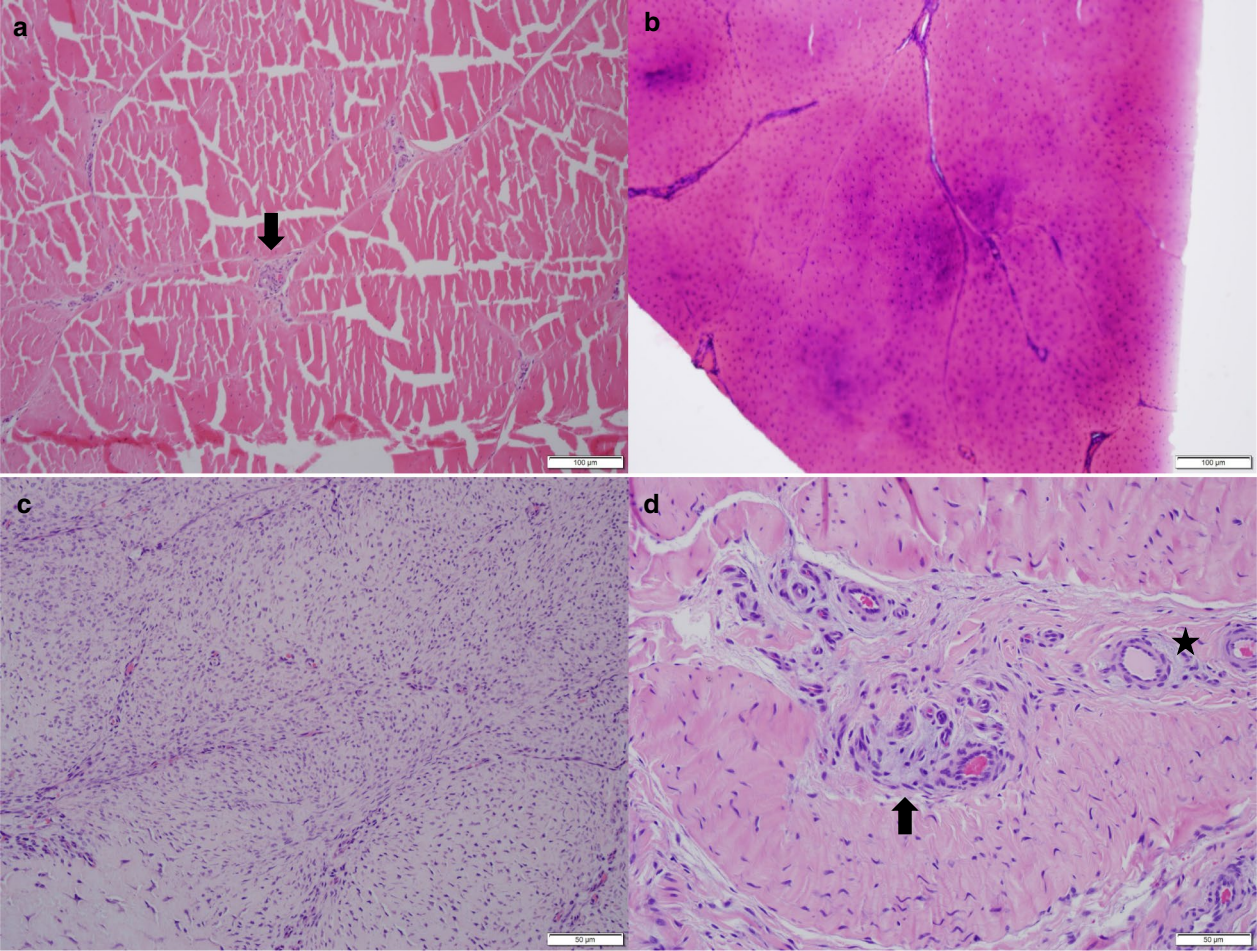
Basic histopathology of DSLD lesions. **a** Histology of normal tendon shows bundles and fascicles separated by septa of less organized and somewhat loose connective tissue that contains fibroblasts, loose collagen fibers, adipose tissue and small blood vessels (↓). **b** A DSLD-affected tendon shows infiltration of proteoglycans (staining dark blue or purple) obscuring the normal architecture of the tendon. **c** Cellular lesions are visualized as distinct foci consisting of spindly active fibroblasts/tenoblasts, arranged in whorls. The presence of small amounts proteoglycans is limited to the cytoplasm of these cells. **d** Reveals an area of septum with increased number of fibroblasts containing small amount of proteoglycans (↓). Numerous small blood vessels are present as well (*). All four sections are stained with hematoxylin and eosin

Normal tendons, especially those of adult horses, are not very cellular. They consist of thick collagen fibers with few tenocytes scattered within and among fibers. Bundles and fascicles are separated by septa of loose connective tissue containing fibroblasts, loose collagen fibers, adipose tissue and small blood vessels (Fig. 1a). Proteoglycans are present in normal tendons and SLs in small amounts and are identifiable only with special stains, such as alcian blue. One of their roles is to regulate collagen fibrillogenesis [3]. As the basic histopathology of DSLD was described previously [3], we are pointing out only the major points relevant for this study. The hallmark of DSLD are inappropriate acellular foci or accumulations of proteoglycans in tendons and other connective tissues, especially SLs without any evidence of inflammation. Such deposits are clearly recognizable even with hematoxylin-eosin staining as blue to purple acellular material (Fig. 1b) and likely represent a more advanced stage of DSLD, particularly when cartilage metaplasia and occasional foci of calcification are present.

In addition, we have observed two types of increased cellularity in some cases of DSLD. The first type forms distinct foci in affected tendons and SLs, consisting of spindly, plump active fibroblasts/tenoblasts, sometimes arranged in whorls and containing small amounts of cytoplasmic proteoglycans (Fig. 1c). The foci were not associated with what we consider more established areas of DSLD (though they may co-exist in the same tendon) described above. However, even in the more advanced stage of DSLD increased cellularity was observed in septa separating bundles and fascicles of quite a few tendons and SLs (Fig. 1d), especially in younger horses. The cells were fibroblasts arranged in sheets. On occasion their cytoplasm also contained small amount of proteoglycans. Proliferation of small blood vessels containing proteoglycans in their media at times was noted as well (Fig. 1d).

#### Presence of TGFβ-1 and BMP2

**Fig. 2:**
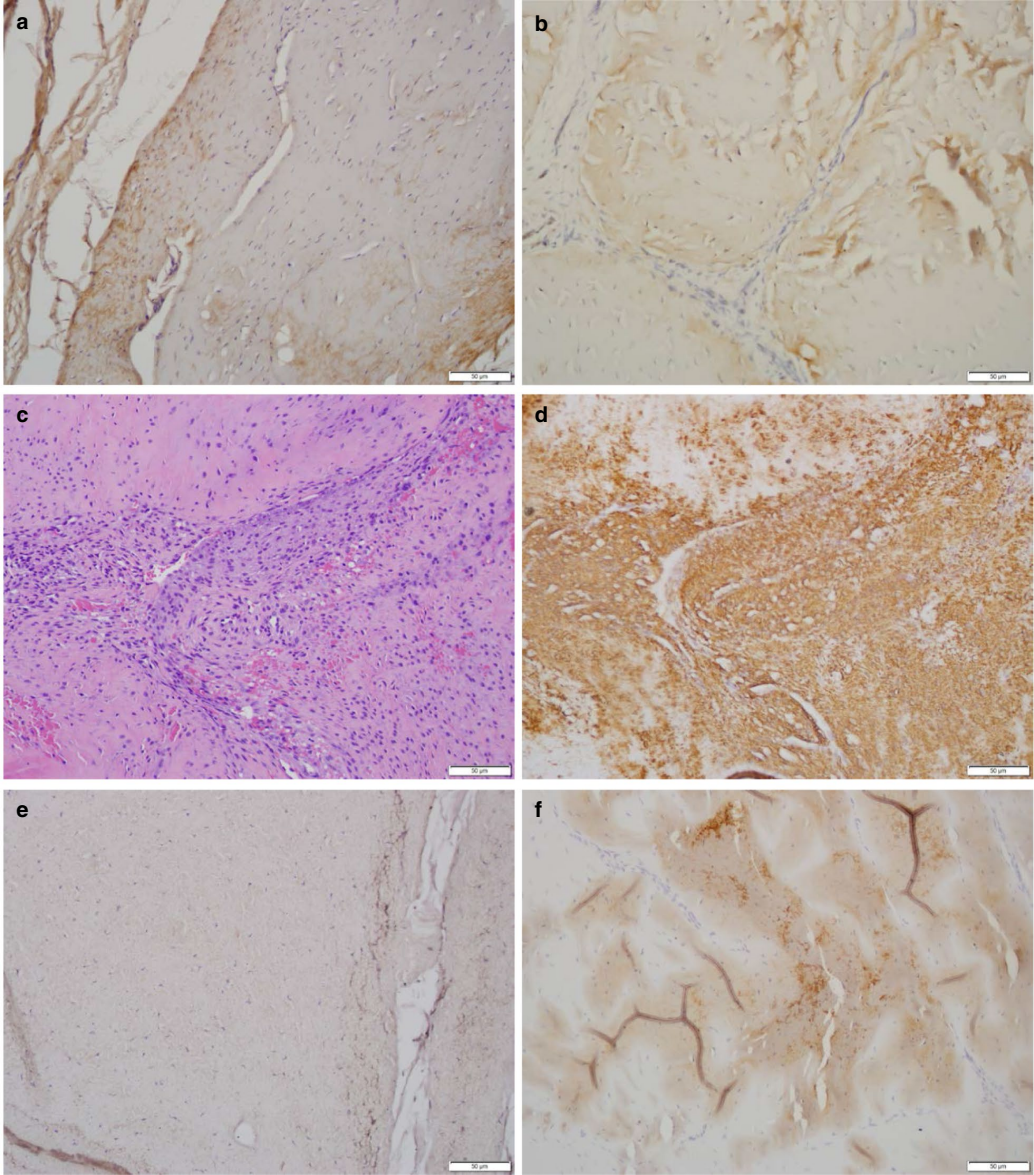
Immunohistochemical visualization of TGFβ-1 and BMP2. Using immunohistochemistry only little TGFβ-1was visualized in fibroblasts and tenoblasts, in both control (**a**) and DSLD-affected (**b**) tendons. **c** Reveals hematoxylin and eosin stained cellular foci and whorls in DSLD-affected tendons. **d** The cells in these foci immunostained strongly for BMP2. No or only little BMP2 was present in acellular areas or masses containing proteoglycans (**e**) and likewise no or very little BMP2 was identified in cells of the control tendons (**f**)

Of the numerous growth factors participating in normal physiology of a tendon TGFβ-1 is probably the most potent stimulant of synthesis of components of extracellular matrix, including type I collagen, the crucial structural constituent of tendons and, to lesser extent, SLs [15]. However, the presence of cartilaginous like material in DSLD-affected tendons and SLs indicates that other, chondrogenic growth factors might be instrumental in DSLD pathogenesis, leading to the accumulation of proteoglycan masses and cartilage metaplasia. BMP2 is one of such growth factors. TGFβ-1 as a potent stimulator of collagen production should also be present in tendons and ligaments. Using immunohistochemistry we have identified only mild amount of TGFβ-1 in both control and DSLD-affected tendon and ligaments (Table 1, Fig. 2a, b). This could be because collagens have long half life and so the turnover would be rather slow with no need for TGFβ-1 to be very active and/or overexpressed. TGFβ-1 was present in fibroblasts and tenocytes, but not in collagen fibers of both normal and DSLD-affected tissues or in the proteoglycan masses (Fig. 2a, b). Immunohistochemistry showed increased levels of BMP2 in cytoplasm of tenoblasts forming cellular foci and whorls in DSLD-affected tissues (Fig. 2d). The acellular areas or masses containing proteoglycans showed no or only little BMP2 staining, and if present staining for BMP2 was limited to cells surrounding proteoglycan mass (Fig. 2e). No or very little BMP2 was identified in the control tissues (Fig. 2f). One section of a tendon from C4 control horse revealed a small area where cells with normal morphology and arrangement stained moderately for BMP2.

Immunohistochemistry identified both TGFβ-1 and BMP2 in tendon sheath and coverings like peritenon and epitenon in all examined samples in moderate amounts regardless of the presence of DSLD.

#### Epimerase presence

That dermatan sulfate epimerase was identified in both healthy and DSLD-affected tissues is not surprising as it is required for proper formation of dermatan sulfate, a GAG chain complexed with decorin protein core. Immunohistochemistry revealed only mild staining in tenoblasts of both normal and DSLD-affected tendons and SLs.

### Discussion

Until recently, equine DSLD was considered to be a collagen disorder localized to SLs of distal limbs of horses. We have shown that not only it is a systemic disorder, but that the primary problem is an excessive accumulation of proteoglycans [3]. We have identified altered glycosylation of decorin GAG chain in which chondroitin sulfate is substituted for the usual dermatan sulfate [5]. We hypothesized that changes in dermatan sulfate epimerase might be responsible for the abnormal substitution and that increased activity of a chondrogenic growth factor(s) may play roles in pathogenesis of DSLD [5]. To further elucidate this mechanism we have investigated the presence of BMP2, a chondrogenic growth factor, TGFβ-1, a potent stimulator of collagen synthesis, and dermatan sulfate epimerase, an enzyme responsible for conversion glucuronic acid into iduronic acid during synthesis of dermatan/chondroitin sulfate chains in tendons and SLs collected from control and DSLD-affected horses. Our goal was to identify compounds important for the development of inappropriate proteoglycan accumulation and cartilage metaplasia in equine DSLD. The increased presence of BMP2 in cellular foci together with the presence of small amounts of proteoglycans in cytoplasm of these tenoblasts/fibroblasts suggests direct involvement of BMP2 in production of proteoglycans by DSLD-affected tenoblasts. The absence of BMP2 in masses of proteoglycans is not unexpected as these lesions are largely acellular and, in all likelihood, represent late development of DSLD where proteoglycans replace not only cells but also collagen fascicles and bundles.

Little or no BMP2 was found in control tendons and SLs because BMP2 stimulates the formation and proliferation of osteoblasts and chondrocytes rather than tenoblasts, and therefore it is usually not found in normal tendons and SLs. Finding high levels of BMP2 in DSLD tendon and SL tissues supports our hypothesis that the overproduction of proteoglycans could stem from an overabundance of at least one chondrogenic growth factor and confirms findings of BMPs in cases of human patellar tendinopathy characterized by cartilage formation [16]. In DSLD-affected tissues the level of immunostained TGFβ-1 was surprisingly low. This is interesting because TGFβ-1 is well known to have osteoblastic as well as chondrogenic effects on mesenchymal stem cells [15]. TGFβ-1 is known to be present in tendons and ligaments, though its low level of expression in normal and DSLD-affected tendon cells/fibroblasts may be indicative of low metabolic turnover of collagen metabolism in the tendon and SL. This would be in agreement with a recent study showing a decrease in TGFβ-1 signaling target gene expression in adipose-derived stromal fibroblasts in DSLD Peruvian Paso horses [17].

We did expect the levels of dermatan sulfate epimerase to be lower in DSLD tissues because our previous data have shown decreased conversion of glucuronic acid to iduronic acid, and thus for synthesis of chondroitin sulfate rather than dermatan sulfate, a process performed by dermatan sulfate epimerase [5]. However, a mutation in the epimerase gene in DSLD would not be detected by immunohistochemistry.

Therefore, we were not able to determine whether the enzyme is active or whether it underwent mutation in DSLD, and thus a change in activity rather than quantity.

In conclusion, we hypothesize that proteoglycan producing cells, most likely tenoblasts, form foci in active disease and are driven to produce excessive amounts of proteoglycans through secretion of BMP2, and perhaps of other chondrogenic growth factors during progression of DSLD.

## Limitations

- Only immunohistochemistry was used.
- Poor results with in situ hybridization because of strong background staining of acellular proteoglycans.
- The use of antibodies to only a few molecules was due to budget limits.

## Abbreviations

BMP: bone morphogenetic protein
DDFT: deep digital flexor tendon
DSLD: degenerative suspensory ligament desmitis
GAG: glycosaminoglycan
RT: room temperature
SDFT: superficial digital flexor tendon
SL: suspensory ligament
TGFβ: transforming growth factor β
